# Concerted phenotypic flexibility of avian erythrocyte size and number in response to dietary anthocyanin supplementation

**DOI:** 10.1186/s12983-023-00487-y

**Published:** 2023-02-24

**Authors:** Maciej Dzialo, Amadeusz Bryła, Kristen J. DeMoranville, Katherine M. Carbeck, Olivia Fatica, Lisa Trost, Barbara Pierce, Edyta T. Sadowska, Scott R. McWilliams, Ulf Bauchinger

**Affiliations:** 1 Faculty of Biology, Institute of Environmental Sciences, Jagiellonian University, 30-387, Kraków, Poland; 2 Department of Natural Resources Science, University of Rhode Island, Kingston, RI 02881 USA; 3 Department of Forest and Conservation Sciences, University of British Columbia, Vancouver, BC V6T1Z4 Canada; 4 Department of Biology, Sacred Heart University, Fairfield, CT 06825 USA; 5 Department for Behavioural Neurobiology, Max Planck Institute for Ornithology, 82319 Seewiesen, Germany; 6 Nencki Institute of Experimental Biology, Polish Academy of Sciences, 02-093 Warsaw, Poland

**Keywords:** Anthocyanin supplementation, Antioxidant diet, Cell number, Cell size, Erythrocytes, Phenotypic flexibility, Red blood cells

## Abstract

**Background:**

Endurance flight impose substantial oxidative costs on the avian oxygen delivery system. In particular, the accumulation of irreversible damage in red blood cells can reduce the capacity of blood to transport oxygen and limit aerobic performance. Many songbirds consume large amounts of anthocyanin-rich fruit, which is hypothesized to reduce oxidative costs, enhance post-flight regeneration, and enable greater aerobic capacity. While their antioxidant benefits appear most straightforward, the effects of anthocyanins on blood composition remain so far unknown. We fed thirty hand-raised European starlings (*Sturnus vulgaris*) two semisynthetic diets (with or without anthocyanin supplement) and manipulated the extent of flight activity in a wind tunnel (daily flying or non-flying for over two weeks) to test for their interactive effects on functionally important haematological variables.

**Results:**

Supplemented birds had on average 15% more and 4% smaller red blood cells compared to non-supplemented individuals and these diet effects were independent of flight manipulation. Haemoglobin content was 7% higher in non-supplemented flying birds compared to non-flying birds, while similar haemoglobin content was observed among supplemented birds that were flown or not. Neither diet nor flight activity influenced haematocrit.

**Conclusion:**

The concerted adjustments suggest that supplementation generally improved antioxidant protection in blood, which could prevent the excess removal of cells from the bloodstream and may have several implications on the oxygen delivery system, including improved gas exchange and blood flow. The flexible haematological response to dietary anthocyanins may also suggest that free-ranging species preferentially consume anthocyanin-rich fruits for their natural blood doping, oxygen delivery-enhancement effects.

**Supplementary Information:**

The online version contains supplementary material available at 10.1186/s12983-023-00487-y.

## Background

Fruits provide frugivores with energy, vitamins and minerals [1, 2] as well as phytochemicals that may yield specific physiological benefits for animals [3]. For example, anthocyanins are pigments responsible for the dark-red colours of fruit and serve as strong free radical scavengers and metal chelators [4–6], that could benefit both the plants and their consumers. Specifically, anthocyanins may either directly mitigate oxidative stress by quenching free radicals and disrupting free radical formation [4], or indirectly affect oxidative balance by activating signalling pathways involved in endogenous antioxidant responses [7, 8]. In addition, anthocyanins are known to improve energy metabolism [9–12], increase vasodilation [13–17], regulate iron metabolism [18, 19], attenuate the endocrine stress response [20], and stimulate reproductive hormones and behaviour during the breeding season [21]. The wide range of health-stimulating actions identifies anthocyanins as a valuable dietary component that could be particularly rewarding during energy-demanding events, when the risk of oxidative stress is considered high [22–25].

The benefits of consuming dietary anthocyanins could be relevant for birds during the migratory season, which is known to impose distinct oxidative and metabolic challenges on migrants [26–31]. For example, during endurance flight birds may operate at five to seven times higher metabolic rate than when at rest [32–35] and this extreme metabolic effort is known to increase generation of reactive oxygen species that could damage tissues and in turn tradeoff aerobic metabolism and performance during migration [28, 36–39]. To minimize the oxidative stress risk, birds, like other vertebrates including humans, can rely on endogenous antioxidants [28, 37, 40] that quench and balance the excess production of reactive oxygen species under most exercise conditions [37, 41]. In addition, birds may also consume dietary antioxidants, which have been proposed to relieve oxidative stress-related costs [42–44]. Specifically, dietary antioxidants, including anthocyanins, may be consumed prophylactically to increase antioxidant capacity or therapeutically to prevent the accumulation of damage in tissues and speed up the recovery following damage [22, 25, 28, 37, 42, 45, 46]. Presumably, the reduction of oxidative costs and enhanced regeneration of metabolically active tissues provided by dietary anthocyanins could enable greater aerobic capacity and thus promote phenotypic adjustments that support more efficient oxygen supply during exercise. However, the effects of dietary anthocyanins on the oxygen delivery system have not yet been investigated.

The oxidative hazard during intense physical activity can be acute to metabolically active tissues such as skeletal muscles, heart [41, 47] and blood [48]. Specifically, red blood cells are vulnerable to oxidative damage because of their continuous exposure to high oxygen fluxes, the high content of heme–iron (a potential catalyst of free radicals formation), and membranes that are rich in polyunsaturated fatty acids [49–52]. Moreover, mature erythrocytes are unable to repair or synthesize damaged proteins de novo [53], hence the oxidative stress might eventually impair the oxygen delivery system by permanent alterations in red blood cell properties and/or by accelerated removal of unfunctional erythrocytes from the bloodstream. The alterations in erythrocytes may include loss of cell volume and surface [54–56]. When cell shrinkage is non-uniform (i.e., involves changes in the shape) and entails a decreased surface-area-to-volume ratio (e.g. due to a greater degree of loss of surface than volume), then it can reduce cell deformability [56–58]. In consequence, this can substantially impede the passage of red blood cells through the microcirculation [59, 60]. In addition, oxidative damage is known to cause haemolysis [54, 61–63], which, if not compensated by increased erythropoiesis, may lead to anaemia that have organism-wide consequences including fatigue and impaired physical performance. In birds, the exercise-induced anaemia could occur during migration as indicated by low haematocrit and haemoglobin content of bar-tailed godwits (*Limosa lapponica taymyrensis*) sampled upon their arrival at a migratory stopover [64, 65]. To prevent the potential oxidative costs in blood, birds may enhance their antioxidant defenses through increased or selective consumption of dietary anthocyanins and other antioxidants, which are known to increase the resistance of erythrocytes to free radical oxidation [61, 66–70].

Despite evidence that dietary anthocyanins are beneficial for antioxidant capacity in avian blood [71], to our best knowledge, their effects on haematological variables have not been investigated in the context of intense and regular physical activity like that associated with continuous flight of migratory birds. Our research study focuses on whether migratory birds can benefit from the consumption of an anthocyanin-rich diet during the energy-demanding event. Specifically, we tested the hypothesis that long-term anthocyanin supplementation and regular flying interactively affect functionally important blood properties in a short-distance migratory bird, the European starling (*Sturnus vulgaris*). We hand-raised thirty hatchling birds and employed a two-factorial experiment that involved manipulation of dietary anthocyanin availability (with or without supplementation) and extent of flying (daily flying or non-flying) to test for their effects on a suite of common haematological variables. Flights were performed during natural spring migration season of starlings living in Germany, in an advanced research wind tunnel that enables precise control of steady flight conditions [32, 72] and has been successfully used in numerous flight-related studies on birds [20, 32, 73]. We predicted that starlings flown each day for several weeks would have reduced red blood cell number, lower haematocrit and haemoglobin content, and smaller cell size compared to more sedentary starlings. We also expected that these flight-induced changes in blood parameters would be mitigated in anthocyanin-supplemented starlings and that the more sedentary, non-flying groups would have similar haematological profiles because of their reduced physical activity and associated lower risk of oxidative stress in blood. Captivity and a sedentary lifestyle in birds can also cause oxidative stress, although it is apparently lower when compared to a more active lifestyle [74]. It is important to note, however, that oxidative stress is not necessarily increased with increased physical activity but rather mitochondrial functioning and increased protonmotive force [75]. Therefore, our prediction that sedentary birds would be better protected from oxidative stress and consequently would have similar haematological profiles as anthocyanin-supplemented birds should be viewed with caution.

## Results

**Fig. 1:**
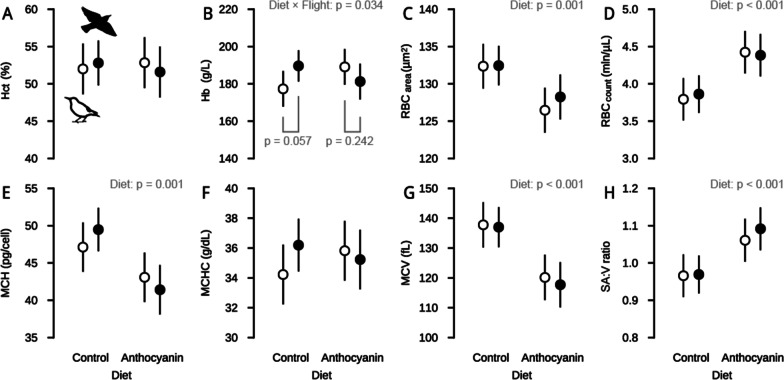
The effect of anthocyanin supplementation and wind-tunnel flight activity on the *primary haematological variables* (upper row) and selected *secondary haematological variables* (lower row). White and black colours represent non-flying and flying birds respectively. Data points are least-squares means (LSM) with 95% confidence intervals (CI) derived from the linear models. *Hct* Haematocrit; *Hb* Haemoglobin content; *RBC*_*count*_ Red blood cell number; *RBC*_*area*_ Red blood cell surface area; *MCH* Mean cell haemoglobin; *MCHC* Mean cell haemoglobin concentration; *MCV* Mean cell volume; *SA:V ratio* Surface-area-to-volume ratio

Out of the four primary haematological variables, only the number of red blood cells (RBC_count_) was increased and the size (RBC_area_) decreased due to diet treatment (Table 1, Fig. 1C, D, Additional file 1: Fig. S1). Haematocrit (Hct) did not respond to any of the experimental treatments (Table 1, Fig. 1A, Additional file 1: Fig. S1), whereas haemoglobin (Hb) content was the only variable for which we detected a statistically significant diet × flight interaction (Additional file 1: Table S1, Fig. 1B, Additional file 1: Fig. S1). Specifically, starlings supplemented with anthocyanin had similar Hb content when flown compared to unflown (*t*_1,25_ = 1.20, *p* = 0.242), whereas starlings not fed the dietary supplement had 7% higher Hb content when flown compared to unflown (*t*_1,25_ = − 2.00, *p* = 0.057). Otherwise, we found clear effects of anthocyanin supplement but not flight on all *secondary* and *shaped-related haematological variables* except mean corpuscular haemoglobin content (MCHC) and red blood cell major axis length (RBC_major_, Table 1, Additional file 1: Fig. S1).

**Table 1:** Results of ANCOVA models for main effects of Diet and Flight and their interaction and Date of sampling as a covariate on haematological variables

Trait	Diet		Flight		Diet × flight		Date of sampling	
	*F*	*p*	*F*	*p*	*F*	*p*	*F*	*p*
Hct	0.014	0.908	0.020	0.890	0.404	0.531	6.898	**0.015**
Hb	0.143	0.708	0.247	0.623	5.026	**0.034**	4.561	**0.043**
RBC_count_	18.494	**< 0.001**	0.011	0.916	0.168	0.685	7.433	**0.012**
RBC_area_	12.794	**0.001**	0.436	0.515	0.362	0.553	0.485	0.493
MCH	15.091	**0.001**	0.048	0.828	1.658	0.210	2.097	0.160
MCHC	0.113	0.740	0.533	0.472	1.841	0.187	1.632	0.213
MCV	26.639	**< 0.001**	0.202	0.657	0.054	0.818	0.245	0.625
SA:V	16.200	**< 0.001**	0.399	0.533	0.257	0.617	0.053	0.821
TSAE	8.945	**0.006**	0.114	0.738	0.044	0.836	5.949	**0.022**
Hb:TSAE	8.478	**0.007**	0.002	0.962	2.556	0.122	1.531	0.228
RBC_major_	0.063	0.803	0.000	0.999	1.480	0.235	0.035	0.852
RBC_minor_	15.549	**0.001**	3.383	0.078	0.025	0.876	0.025	0.877
RBC_ar_	5.584	**0.026**	1.094	0.306	0.737	0.399	0.057	0.813
RBC_circ_	17.305	**< 0.001**	3.253	0.083	0.215	0.647	0.098	0.757

The numerator and denominator degrees of freedom were 1 and 25 respectively for all inference tests

Statistically significant p-values are indicated in bold

*Hct* Haematocrit, *Hb* Haemoglobin content, *RBC*_*count*_ Red blood cell number, *RBC*_*area*_ Red blood cell surface area, *MCH* Mean corpuscular haemoglobin, *MCHC* Mean corpuscular haemoglobin content, *MCV* Mean cell volume, *SA:V* Surface-area-to-volume ratio, *TSAE* Total surface area of erythrocytes, *Hb:TSAE* Haemoglobin-to-TSAE ratio, *RBC*_*major*_ RBC major axis length, *RBC*_*minor*_ RBC minor axis length, *RBC*_*ar*_ RBC aspect ratio, *RBC*_*circ*_ RBC circularity

Anthocyanin supplemented starlings compared to non-supplemented ones had on average 15% higher RBC_count_ (Table 2, Fig. 1D, Additional file 1: Fig. S1) and smaller erythrocyte size indicated by about 4% lower RBC_area_ (Table 2, Fig. 1C, Additional file 1: Fig. S1) and 13% lower mean cell volume (MCV, Table 2, Fig. 1D, Additional file 1: Fig. S1). The reduction of cell size was also associated with about 13% lower mean corpuscular haemoglobin (MCH, Table 2, Fig. 1E, Additional file 1: Fig. S1). Birds fed with anthocyanin-rich diet had increased surface-area-to-volume ratio (SA:V ratio, Table 2, Fig. 1H, Additional file 1: Fig. S1) and total surface area of erythrocytes (TSAE) by about 11% and decreased Hb-content-to-TSAE ratio (Hb:TSAE ratio) ratio by 9%, compared to birds fed with control diet (Table 2, Additional file 1: Fig. S1). In terms of shape, smaller erythrocytes of supplemented birds were more elongated, characterized by a higher aspect ratio (by about 4%) and lower cell circularity (by about 3%), which have resulted from about 3% shorter length of red blood cell minor axis length (RBC_minor_) compared to erythrocytes of non-supplemented birds (Table 2, Additional file 1: Fig. S1). Hct, Hb content, RBC_count_ and TSAE were additionally influenced by the date of sampling (Table 1, Additional file 1: Fig. S1), with all four variables consistently decreasing over the course of the spring season.

**Table 2:** Least Squares Means with 95% confidence intervals of haematological variables of starlings from different diet and flight groups derived from the full ANCOVA models reported in Table 1

Trait		LSM [95% CI] per diet group (averaged across flight activity groups)			LSM [95% CI] per flight activity group (averaged across diet groups)		
		Control	Anthocyanin	% difference relative to control	Non-flying	Flying	% difference relative to non-flying
Hct	Haematocrit (%)	52.40 [50.13–54.67]	52.21 [49.81–54.62]	↓ 0.4	52.42 [50.01–54.82]	52.19 [49.93–54.46]	↓ 0.4
Hb*	Haemoglobin content (mg/cm^3^)	183.46 [177.12–189.81]	185.16 [178.44–191.89]	↑ 0.9	183.20 [176.47–189.93]	185.43 [179.08–191.77]	↑ 1.2
RBC_count_	Red blood cell number (10^6^/mm^3^)	3.83 [3.64–4.02]	4.40 [4.20–4.60]	**↑ 15.0**	4.11 [3.91–4.31]	4.12 [3.93–4.31]	↑ 0.3
RBC_area_	Red blood cell surface area (µm^2^)	132.41 [130.41–134.40]	127.35 [125.24–129.47]	**↓ 3.8**	129.41 [127.30–131.53]	130.35 [128.35–132.34]	↑ 0.7
MCH	Mean cell haemoglobin (pg/cell)	48.30 [46.10–50.50]	42.24 [39.91–44.58]	**↓ 12.5**	45.10 [42.76–47.44]	45.44 [43.24–47.65]	↑ 0.8
MCHC	Mean cell haemoglobin content (mg/mm^3^)	35.21 [33.88–36.54]	35.53 [34.11–36.94]	↑ 0.9	35.02 [33.61–36.44]	35.71 [34.38–37.04]	↑ 2.0
MCV	Mean cell volume (µm^3^)	137.39 [132.33–142.44]	118.92 [113.57–124.28]	**↓ 13.4**	128.96 [123.60–134.32]	127.35 [122.30–132.40]	↓ 1.2
SA:V	Surface-area-to-volume ratio (µm^2^/µm^3^)	0.97 [0.93–1.01]	1.08 [1.04–1.12]	**↑ 11.2**	1.01 [0.97–1.05]	1.03 [0.99–1.07]	↑ 1.7
TSAE	Total surface area of erythrocytes (µm^2^/µm^3^)	253.22 [240.35–266.08]	280.45 [266.81–294.10]	**↑ 10.8**	265.29 [251.65–278.94]	268.38 [255.51–281.25]	↑ 1.2
Hb:TSAE	Haemoglobin-to-TSAE ratio (pg/mm^3^)	0.73 [0.70–0.76]	0.66 [0.63–0.70]	**↓ 9.0**	0.70 [0.66–0.73]	0.70 [0.66–0.73]	↑ 0.2
RBC_major_	RBC major axis (µm)	11.75 [11.56–11.94]	11.79 [11.59–11.99]	↑ 0.3	11.77 [11.57–11.97]	11.77 [11.58–11.96]	0.0
RBC_minor_	RBC minor axis (µm)	7.17 [7.09–7.26]	6.94 [6.85–7.03]	**↓ 3.3**	7.00 [6.91–7.09]	7.11 [7.03–7.20]	↑ 1.6
RBC_ar_	RBC aspect ratio	1.64 [1.61–1.68]	1.70 [1.66–1.74]	**↑ 3.6**	1.69 [1.65–1.72]	1.66 [1.62–1.69]	↓ 1.5
RBC_circ_	RBC circularity	0.81 [0.80–0.81]	0.78 [0.78–0.79]	**↓ 2.7**	0.79 [0.78–0.80]	0.80 [0.79–0.81]	↑ 1.2

Bolded values indicate statistically significant differences between experimental groups—see Table 1 for the results of proper inference tests from ANOVA. ↓ and ↑ arrows indicate respectively decrease and increase response

*Hb content was interactively affected by diet and flight activity; detailed statistics for this interaction and contrast comparisons are described in the main text

## Discussion

Our study provides novel evidence for the effects of anthocyanin supplementation on functionally important haematological variables in songbirds. Long-term supplementation with anthocyanins in starlings resulted in substantially smaller and more numerous red blood cells compared to control birds not fed the anthocyanin supplement. Contrary to our predictions and except for the interaction effect on haemoglobin content, we detected no clear effect of wind tunnel flight activity on the haematological variables. We acknowledge that the unclear flight-induced effects may be due to the potentially mild nature of the exercise, which in turn may not be sufficient to induce oxidative stress and haematological changes in starlings. However, using similar flight training regimens, previous studies have successfully simulated the exercise nature of migratory flights and its effects on bird physiology [20, 32, 76] including the effects on oxidative status [71, 73]. Worth noting, the flying group in our study was exposed to an additional 600–700 km of continuous flapping flight compared to non-flying birds, which likely increases the risk of oxidative damage compared to sedentary birds, although it may not have been sufficient to induce acute effects on haematological variables, such as typically associated with strenuous activities. The observed changes in size and number could have several implications related to the exchange of respiratory gases, blood flow behaviour and efficiency of haemoglobin use.

### Consuming dietary anthocyanins produces smaller and more abundant red blood cells with implications for oxygen delivery

Erythrocytes are continuously exposed to various stressors and oxidative challenges in circulation [48, 52] and thus may particularly require effective antioxidant safeguards [54, 56, 59, 60]. Anthocyanin supplementation has been demonstrated to increase the resistance of red blood cells to oxidative stress [70] and thereby may prevent excessive destruction and removal of erythrocytes from the bloodstream. Alternatively, it is also possible that dietary anthocyanins induce changes in blood independently of their effects on oxidative stress mitigation. For example, anthocyanin supplementation can increase testosterone levels in starlings [21], which in turn can enhance erythropoietic processes in birds (Herrick et al.[77] but see Kern et al. [78]). Considering these various possibilities, we found accordingly that birds fed dietary anthocyanins had about 15% more red blood cells (Fig. 1D) compared to non-supplemented birds. The higher number of erythrocytes in anthocyanin supplemented birds was accompanied by size effects; erythrocytes were with about 4% lower surface area (Fig. 1C) and about 13% lower volume (Fig. 1G) substantially smaller than that of control birds. The adjustments in the two size-related traits resulted in about 11% higher surface-area-to-volume ratio (Fig. 1H). However, it is important to note that the increase in the surface-area-to-volume ratio was due to non-uniform shrinkage, which favorably impacted both cell size and shape (Table 2). Possibly, if non-uniform shrinkage had caused a larger loss of surface area than volume, it could have had a negative effect on oxygen delivery processes through a decrease in the surface-area-to-volume ratio (see next paragraphs). Interestingly, in mammals, senescent erythrocytes appear to decrease cell surface area by a similar degree as cell volume and this may consequently lead to constant surface-area-to-volume ratio and more dense cells [56, 79, 80]. Assuming that similar mechanisms are involved in birds, the considerable increase in surface-area-to-volume ratio found in our study may suggest that the cell size changes not only result from a higher proportion of older cells since they were better protected from oxidative damage, but could be also accompanied by additional structural changes in the cytoskeleton and shape of erythrocytes. The bullet-like shape of erythrocytes (i.e., shorter minor axis, higher aspect ratio, lower circularity, Table 2) found in anthocyanin-supplemented birds might also suggest that the cells are less resistant to flow and may have larger contact area with surrounding tissues [81]. Taken together, the increase in red blood cell number with the paralleled decrease in cell size and adjustments in shape may support the view that circulating erythrocytes of anthocyanin supplemented birds are better protected against oxidative damage. Yet, the physiological significance of changes in cell number, size, and shape of avian erythrocytes requires further investigation.

One of the most important implications of adjustments in the size of red blood cells is the effect on the exchange of respiratory gases. In general, smaller erythrocytes are known to have a higher rate of oxygen uptake and release, which is attributed to a larger surface-area-to-volume ratio of smaller cells [82–84]. However, due to relatively high energy demands for the maintenance of ion gradients, smaller cells may promote higher basal metabolism at the whole animal level [85, 86]. The negative relationship between basal metabolic rate and erythrocyte size has been demonstrated in various vertebrates at the inter- [87–89] and also the intra-specific level [90–94]. High surface-area-to-volume ratio found in anthocyanin-supplemented birds thus could facilitate considerably faster gas exchange, although this might come at higher energy costs required for maintenance of metabolism. However, it is important to note that increased energy metabolism of the cell does not necessarily increase the production of reactive oxygen species, and can actually have the opposite effect depending on the balance between mitochondrial efficiency and uncoupling processes [75]. Further studies should address the question of how red blood cell size and surface-area-to-volume ratio affect mitochondrial functioning and oxidative stress.

In addition to the effects on the exchange of respiratory gases, the decreased cell size and the increased surface-area-to-volume ratio could also promote more efficient blood flow. Specifically, there is a positive relationship between surface-area-to-volume and the degree to which erythrocytes can deform when passing through narrow capillaries [95–97]. Furthermore, the suspensions of smaller red blood cells were found to have lower resistance to flow than that of larger cells [98]. Accordingly, the here observed changes in red blood cell size and surface-area-to-volume ratio may not only suggest the improved erythrocyte deformability but also serve as the mechanism to reduce blood flow resistance [96, 99–101]. Importantly, blood flow can be considerably reduced by high haematocrit, a well-known factor that increases blood viscosity [96, 102–104]. In this study, despite the substantial increase in red blood cell number, haematocrit remained unchanged (Fig. 1A), which might be explained by the concomitant decrease in erythrocyte size (Fig. 1A, G) or an expansion of plasma volume [104, 105]. The maintenance of constant haematocrit can thus represent the additional mechanism of blood viscosity optimization and could help to minimize the cost of blood circulation at a given flow rate [106, 107], promoting more efficient oxygen transport during exercise [108].

The adjustments in cell size and number could also enable more efficient haemoglobin use, especially when birds encounter increased blood flow [109, 110]. Full haemoglobin saturability in the lungs depends on haemoglobin-oxygen affinity [111], the rate of gas exchange [82] and the available exchange surface of erythrocytes relative to the haemoglobin content in the blood (haemoglobin-to-total-surface-area-of-erythrocytes ratio) [109, 110, 112]. A rapid capillary transit associated with increased blood flow may reduce the time required for full haemoglobin saturation, hence, a larger total surface of erythrocytes area per blood volume unit may help to overcome eventual limitations of increased blood flow and enable more efficient haemoglobin use. We found about 11% larger total surface area of erythrocytes among anthocyanin supplemented birds (Table 2, Supplementary Additional file 1: Fig. S1), which combined with relatively small changes in haemoglobin content, also resulted in a 9% lower haemoglobin-to-total surface area of erythrocytes ratio of supplemented birds compared to non-supplemented ones (Table 2, Supplementary Additional file 1: Fig. S1). This may suggest that the full haemoglobin saturation in non-supplemented starlings could be limited by the rapid capillary transit during flight. Kostelecka-Myrcha [109] found that birds, independently of physiological and environmental conditions, have a constant haemoglobin-to-total surface area of erythrocytes ratio. This constancy has been suggested optimal for haemoglobin saturation with oxygen, which in birds takes place under constant conditions due to cross-current gas exchange in their tubular lungs [109, 113]. Our study challenges this view and demonstrates that the haemoglobin-to-total-surface-area of erythrocytes ratio in birds can be modulated by appropriate adjustments in size and number of red blood cells in response to diet. The increase in total surface area of erythrocytes found among anthocyanin supplemented birds could represent one of the mechanisms to overcome limitations in haemoglobin saturability potentially associated with elevated blood flow, but may also compensate for an eventual reduction in the oxygen-carrying capacity of the blood, ensuring the more efficient haemoglobin use during flight.

### No effect of physical activity (flight) on haematological variables

Haematological variables in birds, like in other vertebrates, are portrayed as phenotypically flexible traits that can respond to the increased levels of physical activity. This response may differ depending on the duration of exercise (i.e. short exercise bouts vs periods of repeated exercise) [114] and it was demonstrated that red blood cell number, haemoglobin content and haematocrit decrease after weeks of increased flight activity in zebra finches [114]. Similarly, reduced haematocrit and haemoglobin content was found in bar-tailed godwits (*Limosa lapponica taymyrensis*) at the beginning of migratory stopover [64, 65]. One explanation could be that migrants suffer from haemolytic anaemia incurred by oxidative stress during endurance flights. Alternatively, birds may increase plasma volume—and thus decrease haematocrit—to reduce blood viscosity and costs associated with circulation [115, 116]. Because in our study the changes in blood haemoglobin occurred without a change in haematocrit (Fig. 1A), birds could have altered the amount of haemoglobin within a single erythrocyte, indicated by the analogous change in mean corpuscular haemoglobin concentration. Although we did not find such an effect (Fig. 1F), there is a relatively large uncertainty around the effect size estimates (Additional file 1: Fig. S1), that may suggest we did not have enough statistical power to detect the effect. Collectively, the interaction effect on haemoglobin content and lack of clear effects of flight activity in other haematological variables may suggest that starlings in our study did not experience flight-induced anaemia or had quickly recovered from flights each day during the wind tunnel flight training period.

### Phenotypic flexibility in erythrocyte size and number and its implications for migratory birds

Simultaneous adjustments in red blood cell number, haematocrit and haemoglobin content often occur in response to changes in environmental and physiological conditions [114, 117–119]. Our study shows that such parallel responses are not consistent and might depend on additional changes in other blood-related traits. Importantly, if we would have studied only the routinely measured variables in avian studies—haematocrit (for review see [120]) and haemoglobin content (for review see [121, 122])—we would have missed the response in red blood cell size and number to the dietary anthocyanin manipulation. Considering this potential bias of many studies that have only single variables, comparable phenotypic flexibility in erythrocyte size in concert with cell number changes like that reported here may in fact occur more often in response to other environmental or endogenous conditions. Clearly, more comprehensive investigations on haematological variables beyond the easily obtained measurements are required.

Independent of flight activity and diet, several erythrocyte traits (i.e., red blood cell number, haemoglobin content and haematocrit, total surface area of erythrocytes; Table 1) decreased from February to March. These temporal trends are consistent with seasonal changes of haematological variables documented in other northern hemisphere birds, with higher values of blood-related traits during winter and lower values during spring and summer [123–125]. The observed effect could be associated with genetically preprogrammed seasonal adjustments in body mass and metabolism [126], which are known to affect energy requirements and, consequently, modulate oxygen demands of the tissues [125].

While our study provides evidence that dietary anthocyanins can have a positive impact on blood parameters, measuring the uptake of anthocyanins into the blood and assessing the total antioxidant capacity of the blood or plasma could have provided more comprehensive insights into the effectiveness of the supplemented diet in preventing oxidative damage to the blood cells. Due to limitations in our experimental design, we were unable to measure these parameters in the current study. However, as previously reported in our companion study [71], we found that anthocyanin supplementation can have mixed effects on the oxidative status of birds. Specifically, we found that there were no consistent changes in oxidative status between birds on diets with and without anthocyanin supplement. Starlings on the anthocyanin-rich diet decreased in non-enzymatic antioxidant capacity during the 15-day flight training period and this decrease occurred regardless of whether the birds were trained or untrained. In contrast, birds on the control diet maintained their non-enzymatic antioxidant capacity over the course of this period [71]. These results suggest that anthocyanin-supplemented birds may have utilized non-enzymatic antioxidants more efficiently than birds fed with the control diet. In future studies, it would be beneficial to include measurements of the uptake of anthocyanins into the blood. This would allow for a better understanding of the observed effects. Additionally, assessing the total antioxidant capacity of the blood or plasma would provide more conclusive evidence that the effects seen are due to greater antioxidant protection. Finally, it is important to note that the elderberry extract we used as the supplement contains other phytochemicals in addition to anthocyanins, which can display a range of biochemical effects [127], and thus contribute to the observed changes in haematological parameters. Although we cannot retrospectively measure the total antioxidant capacity and phytochemical profile of the control and anthocyanin-rich diets, we suggest that future studies could include these measurements in order to fully understand the extent of possible diet-treatment effects.

The flexible response of blood composition to dietary anthocyanins is novel and suggests that free-ranging species may preferentially consume anthocyanin-rich fruits for their natural blood doping, oxygen delivery-enhancement effects. Because our study focused exclusively on female birds, it is important to note that further research is needed to assess potential gender-specific differences in response to dietary anthocyanins. Nevertheless, our findings are likely generalizable to males, similar to what was previously observed in the effects of dietary flavonoids (including anthocyanins) on the immune responsiveness of black caps [42]. Although the underlying mechanism, as well as the ecological relevance for this phenotypic flexibility, remains to be directly investigated, the observed adjustments in the size and number of red blood cells may impact several variables associated with the efficiency of supply and removal of respiratory gasses to and from the tissues. A combination of potential effects on the exchange of respiratory gasses, blood flow behaviour and efficiency of haemoglobin use, may set up the physiological machinery for an energy-efficient metabolism. Such slow but enduring effects established through dietary effects could still be modulated by short-term effects, for example in response to immediate responses like shown for other song birds (e.g., [114]). In other words, an additional decrease in red blood cell size, as well as the increased haematocrit and haemoglobin content at the moment of peak aerobic metabolism, may further enhance oxygen supply or may provide assets against the eventual limitations in the oxygen delivery system during flight [114]. Considering the dietary preferences of many songbirds during the migratory season [26, 27], our study also supports the view that migrants could select anthocyanin-rich fruits for their natural oxygen delivery-enhancements effects.

## Methods

### Hand-raising of hatchlings from a local colony of migratory European starlings

Thirty female five-to-eight-day old European starlings (*Sturnus vulgaris*) were collected from nest boxes in late-April to early-May 2015 from a native colony in Upper Bavaria, South Germany (47° 58′ N, 11° 13′ 142 E) and brought into an animal care facility at the Max Planck Institute for Ornithology (MPIO), Seewiesen, Germany. We chose to focus on female starlings as they may face unique physiological challenges during migration in relation to their reproductive success. Migratory birds prepare to breed shortly after spring migration and depositing antioxidants into eggs has been shown to lead to higher hatching and fledging rates for offspring [128, 129]. Female birds may thus face substantial oxidative tradeoff during migration, as they must balance preventing oxidative damage with their future reproductive success [44]. Hatchlings were hand-raised and fed a high protein diet consisting of bee larvae, crickets, wax worms, green bottle fly larvae (pinkies and buffaloes), beef heart with vitamin mixture and calcium carbonate powder. Once hatchlings were able to feed independently, we additionally offered them live mealworms, fresh fruits and vegetables. The diet has been successfully used to hand-raise starlings at the MPIO in previous experiments [32, 73, 130]. From about the age of 35–100 days, the starlings were moved to outdoor aviaries, kept under a natural light cycle, and maintained on an MPIO diet that consisted of insect powder, lettuce, fresh apples and oranges, dried fruit pellets, and mealworms.

### Experimental diets and supplementation with dietary anthocyanins from elderberry fruits

Starting from August 2016, birds were fed with a semi-synthetic (agar-based) diet that simulated a relatively high lipid natural fruit diet (41% carbohydrates: 23% protein: 20% fat) [1, 131]. In October 2016, starlings were randomly assigned to either control-diet (*n* = 16) or anthocyanin-diet (*n* = 14) group. The two semi-synthetic diets were identical except 1.64 mg of standardized elderberry extract (6.5% Powder; Artemis International, Inc., Fort Wayne, IN, USA) per gram of food was included in the anthocyanin diet (see [21] for the detailed composition of these diets). Elderberries are one of the most anthocyanin-rich fruits found in nature [132] and are commonly consumed by starlings and other passerine birds during autumn [133]. The anthocyanin concentration was chosen to reflect a natural level of consumption that birds would experience during periods of lower fruit intake [42]. This was determined to be equivalent to a bird consuming around 17 berries per day, based on a daily food intake of 35 g. A standard vitamin-mineral mix was also included in both diets to ensure the nutritional adequacy of other micronutrients and to provide the necessary Vitamin C for efficient use of the dietary anthocyanin supplement [21].

### Housing conditions prior to the start of flight-training

After ca. 100 days old, birds were provided ad libitum fresh semi-synthetic diet and drinking and bathing water each day. From August 2016 to at least February 9, 2017, birds were housed in separate outdoor aviaries (two aviaries per each experimental diet) in groups of 15–20 individuals (the number includes other starlings fed with the same diet, that were used in study by [71]. Outdoor aviaries were 3.0 × 4.0 × 2.0 m with a 3 m long half-wall subdividing the aviary into sections: one larger (1.9 × 4.0 m) and one smaller (1.0 × 4.0 m). During the colder winter months (November to February), food and water were placed on a heater mat to avoid its freezing. The outdoor aviaries were maintained on a 13D:11N light cycle, until December 22, 2016, when the light schedule was temporarily changed (until February 14, 2017) to 10D:14N to simulate the natural light cycle of starlings wintering in Rome. After January 21, 2017, the natural light transmitted through the opaque ceiling and front window of each outdoor aviary was longer than the provided aviary lights so thereafter the birds were exposed to natural photoperiod in the outdoor aviaries (for details see [21]).

### Flight training of cohorts in a wind-tunnel and blood sampling

The following experimental procedures and measurements took place during natural spring migration season of starlings. Prior to February 9, 2017, starlings in each of the two diet groups were randomly assigned to cohorts of four–six individuals each of which included at least two–three birds to be flown in the wind tunnel or not flown. Starting on February 9, 2017, and continuing every three–nine days, birds from a randomly selected cohort were transferred to the wind tunnel facility and housed in indoor aviaries (1.5 × 2.5 × 2.5 m) that surrounded the wind tunnel. These indoor aviaries were maintained on a 13D:11N light cycle and at relatively constant temperature (18–22 °C). Non-flying birds in a given cohort were placed in cloth-covered cages (25 × 50 × 30 cm) with perch and without access to food and water while flying birds were flight trained each day for 15 consecutive days in the wind tunnel. Each day during flight training, flown birds from a given cohort were allowed to fly out of their aviary, into an enclosed area adjacent to the wind tunnel, and then into the working section (L × W: 2 m × 1.2 m; octagon-shaped). Wind speed was a constant 12 m s^−1^ and ambient temperature was 18–22 °C for all experimental flights. Numerous other recent studies have used a similar wind speed and temperature, and a 15-day flight-training regime to successfully fly small groups of starlings for relatively long durations [20, 32, 71, 73, 76, 134, 135]. After five days of habituation to the wind tunnel, all flown birds were exposed to the same 15-day flight-training regime as follows: days 1–3 = 20 min; day 4 = 30 min; day 5 = 60 min; day 6 = 30 min; day 7 = 60 min; day 8 = 90 min; day 9 resting = no flight; day 10 = 120 min; day 11 = 180 min; day 12 resting = no flight; day 13 = 60 min; day 14 = 30 min. On day 15, starlings flew for up to 6 h until they refused to stay aloft and attempted to perch on the ground or netting at least three times within a five-min period.

After the 15 day flight-training period, females in each cohort were transferred to outdoor aviaries that contained seven–eight male starlings (sex ratio of 1.5 males: 1 females) as part of a companion study focusing on the effects of dietary antioxidants on breeding behavior [21]. For the next five consecutive days, females could interact with males while having constant access to their original diet. In the afternoon (~ 15:00) of day 20, birds were transferred back to the wind tunnel facility for overnight measurements of basal metabolic rate (BMR) at 25 °C temperature (data not covered in this paper). Food was removed at ~ 15:00 on Day 20 so that BMR was measured in post-absorptive birds. At ~ 8:00 on day 21, we sampled ~ 400 µL of blood from the brachial vein of each bird using heparinized capillary tubes after puncture with a 17G needle. Blood samples were immediately transferred to a 0.5 mL heparinized Eppendorf tube for temporary storage. Collecting blood samples 5 days after two weeks of daily flying and with males present mimics the arrival to the breeding grounds following the completion of spring migration.

### Measurements of haematological variables

Measurements of haemoglobin (Hb) content, haematocrit (Hct) and red blood cell count (RBC_count_), as well as preparations of blood smears for measurements of red blood cells surface area (RBC_area_), were done within 30–60 min of blood sampling each bird, hereafter referred as *primary haematological variables*. Haemoglobin content (g/L) was measured with HemoCue AB (Angelholm, Sweden) and for the reliability of this method, we additionally cross-calibrated the device with reference to Drabkin’s solution method. To assess Hct, we filled micro-capillaries with ~ 9 µL of blood and then centrifuged them for 6 min at 11,500 rpm (Eickemeyer, Tuttlingen, Germany) and then measured with callipers the fraction of red blood cells to the nearest hundredth of a millimetre. RBC _count_ (per µL) was counted using a standard microscope and Bürker’s chambers. If possible, Hb, Hct and RBC_count_ were measured in duplicate subsamples to increase reliability and the mean was used as estimates of each variable. To measure RBC_area_ (µm^2^) we fixed and stained blood smears on glass slides (staining with Gill II Hematoxylin, Sigma, Aldrich, Germany; Eosin Y, Analab, Warszawa, Poland). Microscope photographs were taken four–five months after preparation of glass slides, under 100-fold magnification of a light microscope (Nikon Eclipse 801, Tokyo, Japan) connected to a camera (Nikon DS-Ri2, Tokyo, Japan) and NIS Elements software (Nikon, Tokyo, Japan). Finally, we used Fiji software (imagej.nig.gov/ij; fiji.sc/Fiji) to measure area from 50 erythrocytes and the mean value was used as an estimate of RBC_area_. The software was set to automatically measure the cell area after image thresholding. Cells with a distorted shape and those deformed due to touching other cells were excluded from the measurements. In addition to cell surface area, the software also measured length (RBC_major_) and width of the cells (RBC_minor_) and calculated the aspect ratio (RBC_ar_; the ratio of major to minor axis) and cell circularity (RBC_circ_; 4π × area × perimeter^–2^; a value of 1 indicates a perfect circle cell shape and as the value approaches 0, cells are increasingly elongated in shape), which we refer to *shape-related variables*. Based on the four primary haematological variables, we calculated a set of *secondary haematological variables* that could help us in the interpretation of eventual alterations in erythrocyte properties: Mean Corpuscular Haemoglobin Content (MCHC, pg/cell) calculated from Hb and RBC_count_, Mean Corpuscular Haemoglobin (MCH, g/dL) calculated from Hb and Hct and Mean Corpuscular Volume (MCV, fL) calculated from Hct and RBC_count_. The surface-area-to-volume ratio of erythrocytes (SA:V ratio, µm^2^/µm^3^) was calculated by dividing the RBC_area_ by MCV, while the total surface area of erythrocytes (TSAE, µm^2^/µm^3^) by multiplying SA:V by RBC_count_. The haemoglobin-to-total-surface-area-of-erythrocytes ratio was calculated by dividing Hb content by TSAE (Hb:TSAE ratio, pg/mm3).

### Statistical analysis

Statistical analyses were performed in R (version 4.0.3, R Core Team 2020). We used linear models to test the effects of diet (control-diet vs anthocyanin-diet), migratory flight activity (non-flying vs flying), and their interactive effect on all haematological variables measured or calculated in this study. To control for potential temporal changes, all models included the date of sampling as a fixed covariate. Initially, all the models contained first and second-order interactions between the covariate and main effects; however, all these interactions were not significant and were removed from the final models. We checked the assumptions of normality, homogeneity and lack of remaining patterns on residuals. To infer the fixed effects, we used Type III *F*-test from the R package “car” (function “Anova”; [136]). We used the R package “emmeans” [137] to estimate (a) the Least Squares Means with 95% confidence intervals for each experimental group (conditional and marginal means), and (b) the standardized effect sizes (Cohen’s *d* [138]; function “eff_size”) following Nakagawa and Cuthill [139]. In the case of statistically significant interaction between main effects, we further analyzed simple effects (Tukey-adjusted) of flight activity (non-flying vs flying) separately within the two diet groups.

## Supplementary Information

**Additional file 1: Fig. S1.** Standardized effect sizes in response to anthocyanin-rich diet among non-flying (**A**) and flying starlings (**B**); in response to wind-tunnel flight activity among control-(**C**) and anthocyanin-diet (**D**) fed starlings. Effect size higher or lower than 0 indicates respectively higher or lower values among anthocyanin supplemented (panel A and B) or flying birds (panel C and D). Circles and squares depict directly measured and calculated variables respectively. Colors: light grey, dark grey and black indicate small (d ≥ 0.2), medium (d ≥ 0.5) and large effect (d ≥ 0.8) sizes following Cohen [138]. Standardized effect size (Cohen’s d) are reported with their 95% confidence interval. The detailed effect size estimates are described in the Additional file 1: Table S1. *Hct* Haematocrit; *Hb content* Haemoglobin content; *RBCcount* Red blood cell number; *RBCarea* Red blood cell surface area; *MCH* Mean cell haemoglobin; *MCHC* Mean cell haemoglobin concentration; *MCV* Mean cell volume; *SA:V ratio* Surface-area-to-volume ratio; *TSAE* Total surface area of erythrocytes; *Hb:TSAE ratio* Haemoglobin-to-total-surface-area-of-erythrocytes ratio. *RBCmajor* Red blood cell major length; *RBCminor* Red blood cell minor axis length; *RBCar* Red blood cell aspect ratio; *RBCcirc* Red blood cell circularity. **Table S1.** Effect size estimates (% change, absolute change and standardized Cohen’s d) for the comparisons of experimental groups (conditional effects) for all haematological variables considered in this study. *Hct* Haematocrit; *Hb content* Haemoglobin content; *RBCcount* Red blood cell number; *RBCarea* Red blood cell surface area; *MCH* Mean cell haemoglobin; *MCHC* Mean cell haemoglobin concentration; *MCV* Mean cell volume; *SA:V ratio* Surface-area-to-volume ratio; *TSAE* Total surface area of erythrocytes; *Hb:TSAE ratio* Haemoglobin-to-total-surface-area-of-erythrocytes ratio. *RBCmajor* Red blood cell major length; *RBCminor* Red blood cell minor axis length; *RBCar* Red blood cell aspect ratio; *RBCcirc* Red blood cell circularity.

## Data Availability

We agree to archive the data associated with this manuscript should the manuscript be accepted. We expect to archive the data at Dryad Digital Repository.
