# Supplementary material for: Concerted phenotypic flexibility of avian erythrocyte size and number in response to dietary anthocyanin supplementation
Source: Front Zool. 2023 Feb 24;20:9. doi: 10.1186/s12983-023-00487-y (PMC9951440; doi:10.1186/s12983-023-00487-y)
Supplement: Supplementary file 1 — Additional file 1: Fig. S1. Standardized effect sizes in response to anthocyanin-rich diet among non-flying (A) and flying starlings (B); in response to wind-tunnel flight activity among control-(C) and anthocyanin-diet (D) fed starlings. Effect size higher or lower than 0 indicates respectively higher or lower values among anthocyanin supplemented (panel A and B) or flying birds (panel C and D). Circles and squares depict directly measured and calculated variables respectively. Colors: light grey, dark grey and black indicate small (d ≥ 0.2), medium (d ≥ 0.5) and large effect (d ≥ 0.8) sizes following Cohen [138]. Standardized effect size (Cohen’s d) are reported with their 95% confidence interval. The detailed effect size estimates are described in the Additional file 1: Table S1. Hct Haematocrit; Hb content Haemoglobin content; RBCcount Red blood cell number; RBCarea Red blood cell surface area; MCH Mean cell haemoglobin; MCHC Mean cell haemoglobin concentration; MCV Mean cell volume; SA:V ratio Surface-area-to-volume ratio; TSAE Total surface area of erythrocytes; Hb:TSAE ratio Haemoglobin-to-total-surface-area-of-erythrocytes ratio. RBCmajor Red blood cell major length; RBCminor Red blood cell minor axis length; RBCar Red blood cell aspect ratio; RBCcirc Red blood cell circularity. Table S1. Effect size estimates (% change, absolute change and standardized Cohen’s d) for the comparisons of experimental groups (conditional effects) for all haematological variables considered in this study. Hct Haematocrit; Hb content Haemoglobin content; RBCcount Red blood cell number; RBCarea Red blood cell surface area; MCH Mean cell haemoglobin; MCHC Mean cell haemoglobin concentration; MCV Mean cell volume; SA:V ratio Surface-area-to-volume ratio; TSAE Total surface area of erythrocytes; Hb:TSAE ratio Haemoglobin-to-total-surface-area-of-erythrocytes ratio. RBCmajor Red blood cell major length; RBCminor Red blood cell minor axis length; RBCar Red blood cell aspect ratio; RBC [file 12983_2023_487_MOESM1_ESM.pdf]

SUPPLEMENTARY MATERIALS

Table S1. Effect size estimates (% change, absolute change and standardized Cohen's *d*) for the comparisons of experimental groups (conditional effects) for all haematological variables considered in this – study. Abbreviations: Hct=Haematocrit; Hb content=haemoglobin content; RBC_{count}=red blood cell number; RBC_{area}=red blood cell surface area; MCH=mean cell haemoglobin; MCHC=mean cell haemoglobin concentration; MCV=mean cell volume; SA:V ratio=surface-area-to-volume ratio; TSAE=total surface area of erythrocytes; Hb:TSAE ratio=haemoglobin-to-total-surface-area-of-erythrocytes ratio. RBC_{major}=red blood cell major length; RBC_{minor}=red blood cell minor axis length; RBC_{ar}=red blood cell aspect ratio; RBC_{circ}=red blood cell circularity.

Non-flying birds<br>Comparison: Anthocyanin Diet vs Control Diet			
Variable	% change [95% CI]	Absolute change [95% CI]	Cohen's *d* [95% CI]
Hct (%)	1.60 [-7.18 – 11.61]	0.83 [-3.98 – 5.65]	0.19 [-0.91 – 1.29]
Hb (mg/cm^3)	6.64 [-0.91 – 15.04]	11.77 [-1.69 – 25.24]	0.96 [-0.17 – 2.10]
RBC_{count} (10^6/mm^3)	16.62 [5.62 – 29.40]	0.63 [0.23 – 1.03]	1.73 [0.52 – 2.94]
RBC_{area} (µm^2)	-4.46 [-7.49 – -1.29]	-5.90 [-10.14 – -1.67]	-1.54 [-2.72 – -0.35]
MCH (pg/cell)	-8.59 [-17.30 – 1.44]	-4.05 [-8.72 – 0.63]	-0.95 [-2.09 – 0.18]
MCHC (mg/mm^3)	4.66 [-3.40 – 13.73]	1.60 [-1.23 – 4.42]	0.62 [-0.49 – 1.74]
MCV (µm^3)	-12.80 [-19.51 – -5.30]	-17.63 [-28.36 – -6.90]	-1.81 [-3.03 – -0.59]
SA:V (µm^2/µm^3)	9.84 [1.37 – 19.38]	0.10 [0.01 – 0.18]	1.29 [0.13 – 2.46]
TSAE (µm^2/µm^3)	11.62 [0.67 – 24.39]	29.14 [1.82 – 56.45]	1.18 [0.02 – 2.33]
Hb:TSAE (pg/mm^3)	-4.17 [-12.84 – 5.75]	-0.03 [-0.10 – 0.04]	-0.48 [-1.59 – 0.63]
RBC_{major} (µm)	-1.10 [-4.39 – 2.36]	-0.13 [-0.53 – 0.27]	-0.35 [-1.46 – 0.75]
RBC_{minor} (µm)	-3.43 [-5.83 – -0.94]	-0.24 [-0.42 – -0.07]	-1.51 [-2.69 – -0.32]
RBC_{ar}	2.24 [-2.16 – 6.92]	0.04 [-0.04 – 0.11]	0.55 [-0.56 – 1.67]
RBC_{circ}	-2.98 [-4.85 – -1.06]	-0.02 [-0.04 – -0.01]	-1.70 [-2.91 – -0.49]

Flying birds<br>Comparison: Anthocyanin Diet vs Control Diet			
Variable	% change [95% CI]	Absolute change [95% CI]	Cohen's *d* [95% CI]
Hct (%)	-2.29 [-10.29 – 6.68]	-1.21 [-5.74 – 3.33]	-0.28 [-1.32 – 0.76]
Hb (mg/cm^3)	-4.42 [-10.64 – 2.38]	-8.38 [-21.06 – 4.31]	-0.69 [-1.74 – 0.37]
RBC_{count} (10^6/mm^3)	13.48 [3.46 – 24.88]	0.52 [0.14 – 0.90]	1.43 [0.31 – 2.55]
RBC_{area} (µm^2)	-3.17 [-6.07 – -0.16]	-4.20 [-8.19 – -0.21]	-1.09 [-2.18 – -0.01]

MCH (pg/cell)	-16.30 [-23.80 – -7.86]	-8.06 [-12.47 – -3.66]	-1.90 [-3.08 – -0.72]
MCHC (mg/mm^3)	-2.66 [-9.56 – 4.94]	-0.96 [-3.63 – 1.70]	-0.38 [-1.42 – 0.67]
MCV (µm^3)	-14.08 [-20.46 – -7.05]	-19.29 [-29.40 – -9.19]	-1.98 [-3.17 – -0.79]
SA:V (µm^2/µm^3)	12.63 [4.52 – 21.64]	0.12 [0.05 – 0.20]	1.67 [0.52 – 2.81]
TSAE (µm^2/µm^3)	9.91 [-0.15 – 21.40]	25.33 [-0.40 – 51.07]	1.02 [-0.06 – 2.10]
Hb:TSAE (pg/mm^3)	-13.63 [-20.98 – -5.40]	-0.10 [-0.17 – -0.04]	-1.66 [-2.80 – -0.51]
RBC_{major} (µm)	1.69 [-1.53 – 5.05]	0.20 [-0.18 – 0.58]	0.54 [-0.51 – 1.59]
RBC_{minor} (µm)	-3.12 [-5.37 – -0.80]	-0.23 [-0.39 – -0.06]	-1.39 [-2.50 – -0.28]
RBC_{ar}	4.93 [0.59 – 9.53]	0.08 [0.01 – 0.15]	1.18 [0.09 – 2.28]
RBC_{circ}	-2.36 [-4.12 – -0.56]	-0.02 [-0.03 – 0.00]	-1.36 [-2.47 – -0.25]

Control-diet fed birds
Comparison: Flying vs Non-flying birds

Variable	% change [95% CI]	Absolute change [95% CI]	Cohen's *d* [95% CI]
Hct (%)	1.53 [-6.74 – 10.96]	0.80 [-3.74 – 5.33]	0.18 [-0.86 – 1.22]
Hb (mg/cm^3)	6.94 [-0.20 – 14.89]	12.31 [-0.38 – 24.99]	1.01 [-0.07 – 2.09]
RBC_{count} (10^6/mm^3)	1.83 [-7.58 – 12.75]	0.07 [-0.31 – 0.45]	0.19 [-0.85 – 1.23]
RBC_{area} (µm^2)	0.06 [-2.89 – 3.15]	0.08 [-3.91 – 4.07]	0.02 [-1.02 – 1.06]
MCH (pg/cell)	4.99 [-4.07 – 15.42]	2.35 [-2.05 – 6.76]	0.55 [-0.50 – 1.60]
MCHC (mg/mm^3)	5.75 [-1.92 – 14.37]	1.97 [-0.69 – 4.63]	0.77 [-0.29 – 1.83]
MCV (µm^3)	-0.56 [-7.49 – 7.17]	-0.78 [-10.88 – 9.33]	-0.08 [-1.12 – 0.96]
SA:V (µm^2/µm^3)	0.35 [-7.13 – 8.77]	0.00 [-0.07 – 0.08]	0.05 [-0.99 – 1.08]
TSAE (µm^2/µm^3)	1.99 [-7.68 – 13.27]	4.98 [-20.74 – 30.71]	0.20 [-0.84 – 1.24]
Hb:TSAE (pg/mm^3)	5.24 [-3.50 – 15.25]	0.04 [-0.03 – 0.10]	0.61 [-0.45 – 1.66]
RBC_{major} (µm)	-1.38 [-4.47 – 1.87]	-0.16 [-0.54 – 0.22]	-0.45 [-1.49 – 0.60]
RBC_{minor} (µm)	1.41 [-0.94 – 3.83]	0.10 [-0.07 – 0.27]	0.62 [-0.44 – 1.67]
RBC_{ar}	-2.84 [-6.82 – 1.41]	-0.05 [-0.12 – 0.02]	-0.70 [-1.76 – 0.36]
RBC_{circ}	0.86 [-0.94 – 2.72]	0.01 [-0.01 – 0.02]	0.49 [-0.56 – 1.54]

Anthocyanin-diet fed birds
Comparison: Flying vs Non-flying birds

Variable	% change [95% CI]	Absolute change [95% CI]	Cohen's *d* [95% CI]
Hct (%)	-2.36 [-10.78 – 7.22]	-1.25 [-6.06 – 3.57]	-0.29 [-1.39 – 0.82]
Hb (mg/cm^3)	-4.15 [-10.73 – 3.14]	-7.84 [-21.32 – 5.64]	-0.64 [-1.76 – 0.48]
RBC_{count} (10^6/mm^3)	-0.92 [-9.40 – 8.72]	-0.04 [-0.44 – 0.36]	-0.11 [-1.21 – 0.99]
RBC_{area} (µm^2)	1.41 [-1.90 – 4.88]	1.78 [-2.46 – 6.02]	0.46 [-0.65 – 1.58]

MCH (pg/cell)	-3.87 [-13.68 – 7.58]	-1.67 [-6.35 – 3.01]	-0.39 [-1.50 – 0.72]
MCHC (mg/mm^3)	-1.65 [-9.05 – 6.62]	-0.59 [-3.42 – 2.24]	-0.23 [-1.34 – 0.87]
MCV (µm^3)	-2.03 [-10.32 – 7.37]	-2.44 [-13.18 – 8.30]	-0.25 [-1.36 – 0.85]
SA:V (µm^2/µm^3)	2.90 [-4.50 – 11.15]	0.03 [-0.05 – 0.11]	0.42 [-0.69 – 1.53]
TSAE (µm^2/µm^3)	0.42 [-8.74 – 10.95]	1.18 [-26.16 – 28.52]	0.05 [-1.06 – 1.15]
Hb:TSAE (pg/mm^3)	-5.15 [-14.11 – 5.17]	-0.04 [-0.10 – 0.03]	-0.57 [-1.69 – 0.55]
RBC_{major} (µm)	1.40 [-2.00 – 4.96]	0.16 [-0.24 – 0.57]	0.45 [-0.66 – 1.56]
RBC_{minor} (µm)	1.73 [-0.85 – 4.41]	0.12 [-0.06 – 0.30]	0.73 [-0.39 – 1.86]
RBC_{ar}	-0.27 [-4.50 – 4.23]	0.00 [-0.08 – 0.07]	-0.07 [-1.17 – 1.03]
RBC_{circ}	1.51 [-0.48 – 3.55]	0.01 [0.00 – 0.03]	0.83 [-0.30 – 1.96]

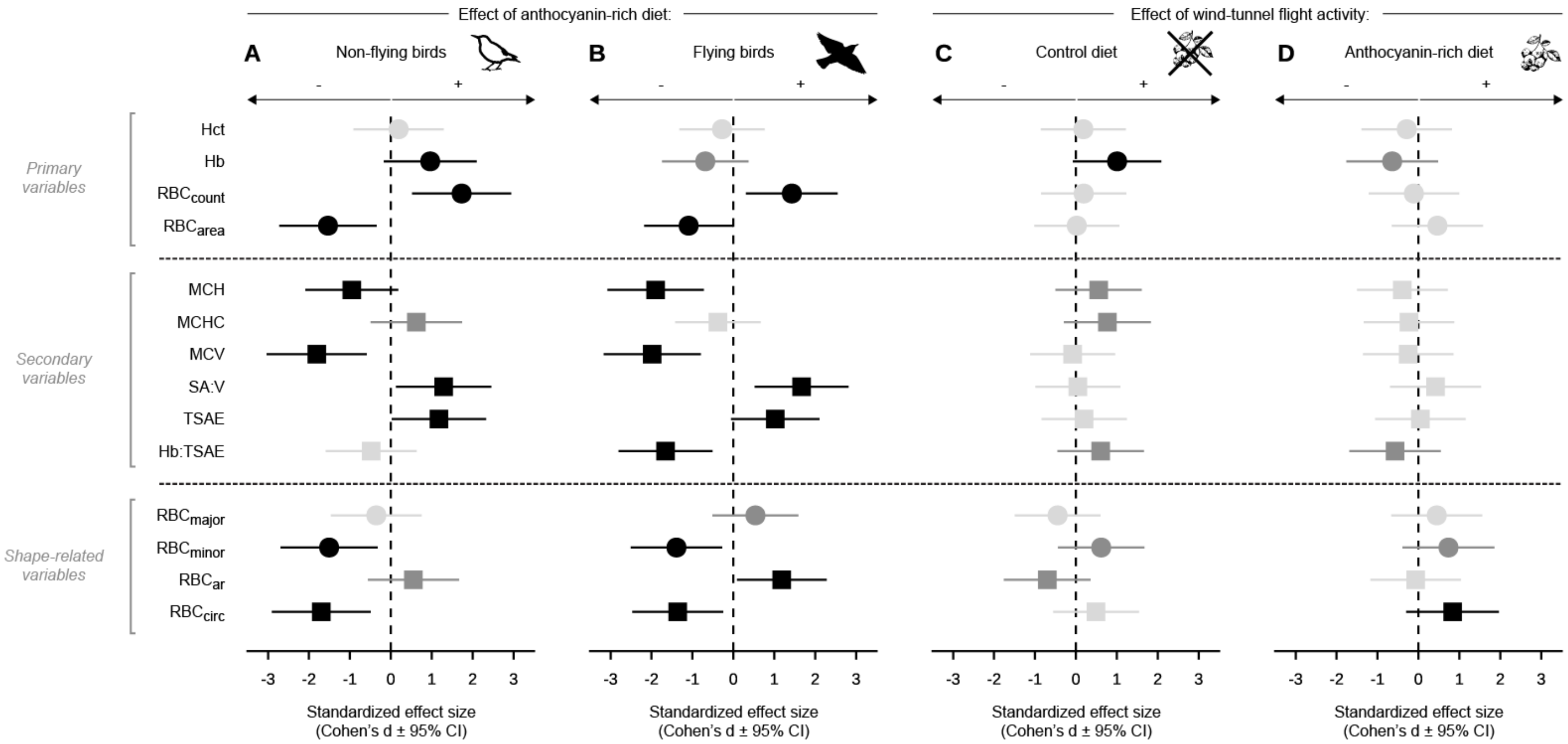

Figure S1. Standardized effect sizes in response to anthocyanin-rich diet among non-flying (A) and flying starlings (B); in response to wind-tunnel flight activity among control- (C) and anthocyanin-diet (D) fed starlings. Effect size higher or lower than 0 indicates respectively higher or lower values among anthocyanin supplemented (panel A and B) or flying birds (panel C and D). Circles and squares depict directly measured and calculated variables respectively. Colors: light grey, dark grey and black indicate small (d≥0.2), medium (d≥0.5) and large effect (d≥0.8) sizes following Cohen (1988). Standardized effect size (Cohen's *d*) are reported with their 95% confidence interval. The detailed effect size estimates are described in the supplementary Table S1. Abbreviations: Hct=Haematocrit; Hb content=haemoglobin content; RBC_{count}=red blood cell number; RBC_{area}=red blood cell surface area; MCH=mean cell haemoglobin; MCHC=mean cell haemoglobin concentration; MCV=mean cell volume; SA:V ratio=surface-area-to-volume ratio; TSAE=total surface area of erythrocytes; Hb:TSAE ratio=haemoglobin-to-total-surface-area-of-erythrocytes ratio. RBC_{major}=red blood cell major length; RBC_{minor}=red blood cell minor axis length; RBC_{ar}=red blood cell aspect ratio; RBC_{circ}=red blood cell circularity.
